# Dissecting structural and nucleotide genome-wide variation in inbred Iberian pigs

**DOI:** 10.1186/1471-2164-14-148

**Published:** 2013-03-05

**Authors:** Anna Esteve-Codina, Yogesh Paudel, Luca Ferretti, Emanuele Raineri, Hendrik-Jan Megens, Luis Silió, María C Rodríguez, Martein AM Groenen, Sebastian E Ramos-Onsins, Miguel Pérez-Enciso

**Affiliations:** 1 Center for Research in Agricultural Genomics (CRAG), Campus UAB, Bellaterra, 08193, Spain; 2 Departament de Ciència Animal i dels Aliments, Universitat Autònoma de Barcelona, Bellaterra, 08193, Spain; 3 Animal Breeding and Genomics Centre, Wageningen University, De Elst 1, Wageningen, 6708 WD, The Netherlands; 4 Centre Nacional d'Anàlisi Genòmica (CNAG), Barcelona, Spain; 5 Departamento de Mejora Genética Animal, INIA, Madrid, 28040, Spain; 6 Institut Català de Recerca i Estudis Avançats (ICREA), Carrer de Lluís Companys 23, Barcelona, 08010, Spain

**Keywords:** Iberian pig, Next generation sequencing, Pig, Selection tests, Structural variation

## Abstract

**Background:**

In contrast to international pig breeds, the Iberian breed has not been admixed with Asian germplasm. This makes it an important model to study both domestication and relevance of Asian genes in the pig. Besides, Iberian pigs exhibit high meat quality as well as appetite and propensity to obesity. Here we provide a genome wide analysis of nucleotide and structural diversity in a reduced representation library from a pool (n=9 sows) and shotgun genomic sequence from a single sow of the highly inbred Guadyerbas strain. In the pool, we applied newly developed tools to account for the peculiarities of these data.

**Results:**

A total of 254,106 SNPs in the pool (79.6 Mb covered) and 643,783 in the Guadyerbas sow (1.47 Gb covered) were called. The nucleotide diversity (1.31x10^-3^ per bp in autosomes) is very similar to that reported in wild boar. A much lower than expected diversity in the X chromosome was confirmed (1.79x10^-4^ per bp in the individual and 5.83x10^-4^ per bp in the pool). A strong (0.70) correlation between recombination and variability was observed, but not with gene density or GC content. Multicopy regions affected about 4% of annotated pig genes in their entirety, and 2% of the genes partially. Genes within the lowest variability windows comprised interferon genes and, in chromosome X, genes involved in behavior like *HTR2C* or *MCEP2*. A modified Hudson-Kreitman-Aguadé test for pools also indicated an accelerated evolution in genes involved in behavior, as well as in spermatogenesis and in lipid metabolism.

**Conclusions:**

This work illustrates the strength of current sequencing technologies to picture a comprehensive landscape of variability in livestock species, and to pinpoint regions containing genes potentially under selection. Among those genes, we report genes involved in behavior, including feeding behavior, and lipid metabolism. The pig X chromosome is an outlier in terms of nucleotide diversity, which suggests selective constraints. Our data further confirm the importance of structural variation in the species, including Iberian pigs, and allowed us to identify new paralogs for known gene families.

## Background

The pig is one of the most important sources of meat worldwide, as well as a relevant biomedical model for some diseases like metabolic syndrome or obesity [1,2]. Current high throughput sequencing technologies, together with the recent completion of porcine’s genome and its annotation [3], makes it possible to study the genomic variability of specific breeds with a detail that was not possible until now. Here, we present a thorough genomewide analysis of the Iberian breed. Commercial pig breeds that are today exploited internationally, e.g., Landrace, Large White or Duroc, are the result of introgressing local primigenious European breeds with Asian germplasm, a process that is now well documented [4,5]. In contrast, European wild boars, as well as local Mediterranean breeds like the Iberian breed, were not affected by this admixture process. Given the high divergence between Asian and primigenious European pigs, ca. 1 MYA [3], and the extent and intensity of modern selection methods, the study of Iberian pigs can illuminate both the domestication process and the influence of Asian germplasm in the shaping of current international pig breeds. Besides, Iberian pigs are important economically because of their high meat quality and resilience to endure harsh environmental conditions [6]. They are very fat pigs, markedly different from modern lean pigs, and are interesting from a human biomedical perspective because they present high feed intake and propension to obesity, compatible with high values of serum leptin [7].

Here, we describe a genomic analysis of the Iberian breed using a mixed approach: a reduced representation library (RRL, [8]) sequencing of a pool of nine sows, and a shotgun complete genome sequencing of a highly inbred Iberian strain (Guadyerbas). The latter strain has been used in numerous QTL experiments and has been maintained in isolation for over 68 years and 25 generations in a closed herd, *El Dehesón del Encinar*, located in Toledo, central Spain [9]. In a previous work [10], we reported a partial RRL sequencing of the same sow, 1% of the genome approximately. The pool is made up of Iberian pigs from farms with strict pedigree control and that represent the extant diversity of Iberian varieties. The pool included as well the Guadyerbas sow that was individually sequenced.

## Results

### Nucleotide variability

Out of two paired-end (PE) lanes from a reduced representation library in the pool, about 3% of the current pig assembly v 10.2 was covered with depth between 3× and 30×. From one PE and one single end (SE) lane of the Guadyerbas sow, ~ 60% of the genome was covered with depths 3× − 20×. Average depths in the pool and in the individual were 14× and 7×, respectively. These statistics result from filtering reads by a minimum mapping quality of 20 with samtools, as suggested to remove ambiguous mapping (http://samtools.sourceforge.net/).

In order to better interpret the results of the pool design and be able to quantify how much variability is likely to be uncovered by sequencing the pool, we ran a simulation study mimicking as much as possible the pool process and the bioinformatics pipeline we used in the analyses of real data (see Materials and methods). These simulations suggested that we should detect ~ 47% of all SNPs actually segregating in the nine individuals and with a low false SNP discovery rate (0.02) for regions covered with a depth of 3-20×. Additional file 1 shows simulated results by minor allele frequency (MAF) and depth. Note that the majority of SNPs that are missed is due to their low frequency: while 80% of SNPs at MAF < 0.1 are likely undetected, the power for SNPs with MAF 0.3 is 60% and approaches 100% at higher MAFs. Importantly, the statistics used here to infer nucleotide variability were developed to account for the bias towards intermediate allele frequency in the pooling process (see Materials and methods).

In all, the raw numbers of SNPs (only segregating sites) called using criteria described in methods were 254,106 in the pool (79.6 Mb covered) and 643,783 in the Guadyerbas sow (1.47 Gb covered). The full SNP list is available on request from the authors. A total of 17.7 Mb of the current assembly was covered in both the pool and the individual, and 10,324 SNPs were called in both designs. The raw number of fixed differences between the assembly, primarily a Duroc female, and the Iberian pool was 152,225, and 2,503,645 for the Guadyerbas. We also detected 49,105 heterozygous indels and 316,189 fixed indels in the individual sow. We did not call indels in the pool because indel calling algorithms are not specific for pools and can be misleading. SNP annotation by autosomes, pseudoautosomal region (PAR) and non-pseudoautosomal region (NPAR) of the X chromosome (SSCX) is detailed in Table 1. SNP classes are ranked in decreasing order of severity of their predicted functional consequences, according to variant effect predictor ensembl pipeline [11]. Note, nevertheless, that these raw numbers are not directly comparable between the pool and the individual because of the (unknown) different number of individuals actually sequenced in the pool in each region, read depth and alignment lengths.

**Table 1:** Number of SNPs by annotation class and genome region: autosomes, X chromosome non-pseudoautosomal region (NPAR) and X pseudoautosomal region (PAR)

**Consequence**	**Autosomes Guadyerbas**	**Autosomes Iberian pool**	**NPAR Guadyerbas**	**NPAR Iberian pool**	**PAR Guadyerbas**	**PAR Iberian pool**
Essential splice site	30	30	1	1	0	0
Stop gained	44	11	2	0	0	0
Stop gained,splice site	1	0	0	0	0	0
Stop lost	4	15	0	0	0	0
Non synonymous coding	2650	1222	40	28	1	0
Non synonymous coding,splice site	51	31	0	1	0	0
Synonymous coding,splice site	49	24	3	0	0	0
Splice site,intronic	282	254	10	8	0	1
5prime utr,splice site	1	1	0	0	0	0
3prime utr,splice site	2	0	0	0	0	0
Within non coding gene,splice site	7	1	0	0	0	0
Synonymous coding	2676	1611	33	34	0	1
Coding unknown	8	4	0	0	0	0
Within mature mirna	1	1	0	2	0	0
5prime utr	193	418	0	7	0	0
3prime utr	2103	1357	23	19	0	0
Intronic	148468	78279	1867	1204	133	147
Within non coding gene	286	99	12	3	0	0
Within non coding gene,intronic	6	3	0	0	0	0
Upstream	34314	15216	426	346	20	16
Downstream	34395	15737	628	346	24	14
Intergenic	433720	150572	7161	3620	3087	1214
Total	659291	264886	10206	5619	3265	1393

Annotation terms shown are in decreasing order of severity, as estimated by Ensembl.

**Figure 1:**
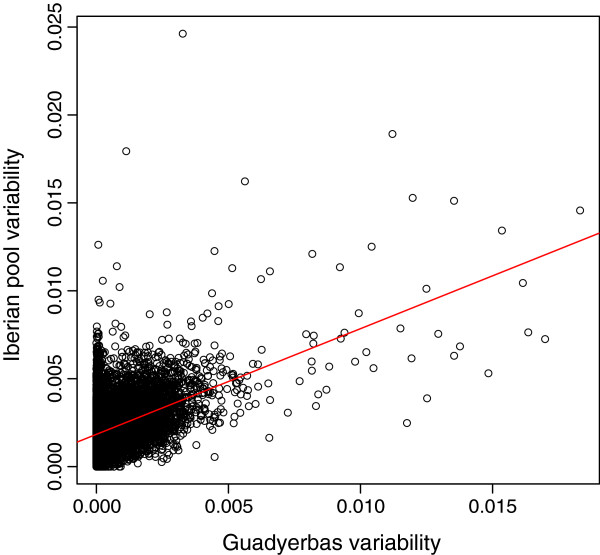
**Correlation of Watterson’s theta estimates of nucleotide variability (per bp) between the individual (Guadyerbas) and the Iberian pool across non overlapping windows of 200 kb length.** Each dot corresponds to theta estimates in individual and pool for the same window.

We computed Watterson’s estimates of diversity, θ, corrected for pooling and low depth (as detailed in methods and in [12]) in non overlapping windows of 200 kb length. In general, there was a moderate correlation between pool and individual variabilities (Pearson correlation = 0.45, Figure 1) when windows with no SNP in the Guadyerbas are removed. Nevertheless, it should be reminded that the Guadyerbas strain is highly inbred, e.g., we found that ~ 10% of the 200 kb windows were devoid of any SNP. Another factor of bias is that, while an RRL was sequenced in the pool (3% of the genome), the Guadyerbas sow was shotgun sequenced (60% genome aligned). Our results suggest a positive correlation in nucleotide diversity among nearby genome regions for the 17.7 Mb that were covered in both the pool and the individual (Figure 1).

**Figure 2:**
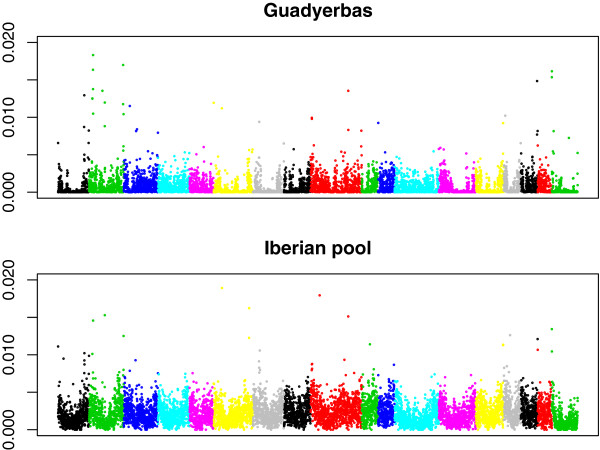
**Watterson’s nucleotide variability (per bp) distribution by chromosome (SSC1-SSC18, SSCX) in the pool (top) and the individual (bottom).** Each dot represents a 200 kb length window, and each chromosome, in a different color.

**Figure 3:**
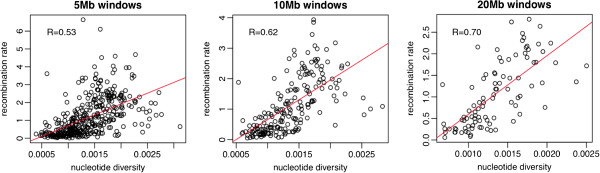
Correlation of Watterson’s theta estimates of nucleotide variability (per bp) in the pool and the recombination rate (cM/Mb) in windows of 5 Mb, 10 Mb and 20 Mb.

Watterson’s θ are plotted in Figure 2 in 200 kb windows for both the pool and the individual. In agreement with results from [12,13] and [10], variability increased towards telomeric regions. This suggests a marked effect of recombination in variability. To explore this issue further, we plotted variability vs. recombination rate [14] in 5Mb, 10Mb and 20Mb window sizes (Figure 3), observing a correlation of 0.53, 0.62 and 0.70, respectively. Correlation increased with window size, probably because the genetic maps were obtained from a pedigree with few generations and therefore small genetic distances are subject to large sampling errors [14]. We also correlated variability with other factors that have been reported to affect variability, namely GC content and gene density [15], and results are in Table 2. Recombination rate was still the main factor affecting variability. Although GC content was also significant, its conditional effect was slightly negative, likely because of colinearity. If a model was fitted with only GC content, the effect was positive although the model explained much lower variability than a model with only recombination rate (results not presented).

**Table 2:** Multiple regression estimates of recombination rate, gene and GC contents on Wattersons' variability estimates (across 20 Mb windows) in the Guadyerbas individual

**Factor**	**Estimate**	**SD**	**P-value**
Recombination rate (cM/Mb)	4.32×10^-4^	4.11×10^-5^	2.00×10^-16^
Average gene length (bp)	−2.17×10^-9^	3.77×10^-9^	0.57
GC content (%)	−3.97×10^-3^	1.12×10^-3^	0.64×10^-3^

In agreement with previous results [10], we observed a marked reduced variability in chromosome X NPAR relative to the expected value, which is 3/4 that of autosomes; this reduction was more pronounced in the inbred Guadyerbas individual than in the pool (Table 3). Note that SSCX is divided in PAR and NPAR regions, which exhibit quite distinct patterns of variability. The high variability regions in the telomeres correspond to the PAR. In fact, variability in the PAR is over 10 times higher than in NPAR for the Guadyerbas sow. Although the porcine PAR is small (~7 Mb) and diversity estimates are subject to larger errors, the difference between PAR and NPAR variabilities is dramatic.

**Table 3:** Nucleotide diversity per bp in autosomes and X chromosome

	**Guadyerbas individual**	**Iberian pool**
Autosomes	6.55×10^-4^	1.31×10^-3^
Pseudo-autosomal chromosome X (PAR)	3.02×10^-3^	2.22×10^-3^
Nonpseudo-autosomal chromosome X (NPAR)	1.79×10^-4^	5.83×10^-4^

### Multicopy regions

Given the increasing awareness of the importance of structural variants in the genome, we also sought to uncover these in the Iberian pigs. In fact, one of the advantages of resequencing vs. genotyping is that the former allows a more precise detection of structural variants in the genome than the latter approach, as discovery of variants with arrays depend on the specific probes used to manufacture the chip. Here, we employed an excess of read density (depth) method to uncover multicopy regions (MCRs, as detailed in methods and in (Paudel Y, Madsen O, Megens H-J, Frantz LAF, Bosse M, Bastiaansen JWM, Crooijmans RPMA, Groenen MAM: Evolutionary dynamics of copy number variation in pig genomes in the context of adaptation and domestication, submitted). We refer to multicopy regions rather than copy number variants because we analyzed a single individual and we do not have information on whether that multicopy region is actually fixed or segregating in the population. The draft status of the current porcine genome assembly does not allow accurate ascertainment of other kinds of variants (e.g., inversions, novel insertions, translocations) using aberrant paired-end distance methods. MCRs detection is based on read density and is therefore less sensitive to mis-assemblies in the reference genome. We analyzed only the individual Guadyerbas sow because of the uncertainty in the number of chromosomes actually sequenced for the pool in any given region. Due to limited read depth, we considered only gains with respect to reference genome rather than gains and losses.

**Figure 4:**
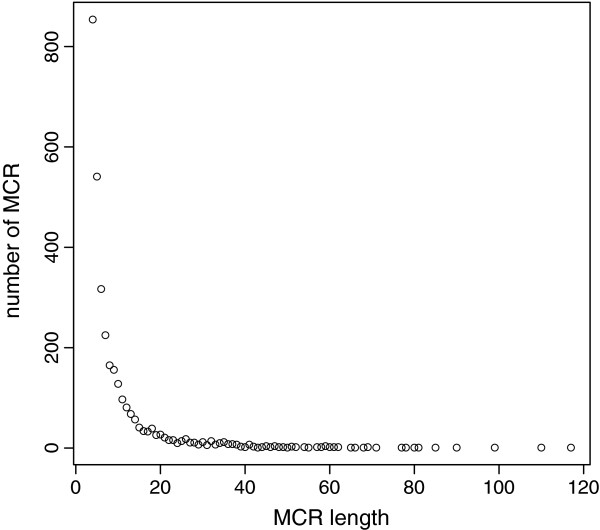
Number of multicopy regions (MCR) by specified length (kb).

**Figure 5:**
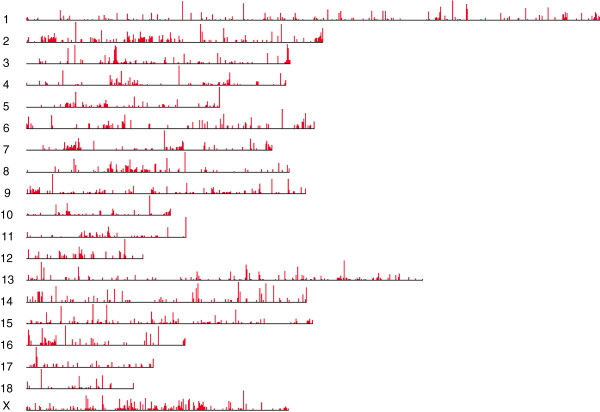
**Chromosome positions of multicopy region gains with respect to the reference genome found in the Iberian genome.** Each vertical red line corresponds to a multicopy region location and the length of the line is proportional to the number of copies. The shortest multicopy region was 4 kb long and the longest, 117 kb.

We found 3,082 outlier regions potentially caused by MCRs in the Guadyerbas genome. These were distributed among 1,653 windows and spanned 30.5 Mb. The majority of the MCR are short (less than 20 kb) and only two are longer than 100 kb (Figure 4). These MCRs affect 4% of the annotated pig genes in their entirety (100% of the gene length) and 2% of the genes partially (>50% of their gene length). Barring for errors in the reference assembly, therefore, MCRs seem to be an important source of variability in the pig, as also observed in other species [16]. Distribution of the MCRs along the chromosomes is represented in Figure 5. We observed a positive correlation between nucleotide variability (Watterson’s θ) inside the MCRs and the nucleotide variability within the 200 kb windows containing MCRs but outside MCR boundaries (Pearson correlation = 0.6, Additional file 2). Average variability inside MCRs was 1.51×10^-3^, somewhat higher than MCR windows but outside MCRs boundaries (9.09×10^-4^), whereas windows devoid of MCRs had the lowest average diversity (8.42×10^-5^), suggesting that windows with high nucleotide variation are enriched in MCRs (Summary statistics in Table 4). On the other hand, we detected no correlation between the number of copies of a MCR and variability within MCRs.

**Table 4:** Nucleotide variabilities (SNPs / bp) within and outside multicopy regions (MCRs) in the Guadyerbas individual

**Region**	**Median**	**Mean**	**SD**
Within MCRs	1.67×10^-4^	1.52×10^-3^	3.46×10^-3^
Outside MCRs, within windows containing MCRs	1.83×10^-4^	9.10×10^-4^	1.82×10^-3^
Windows without MCRs	8.43×10^-5^	3.92×10^-4^	6.11×10^-4^

**Figure 6:**
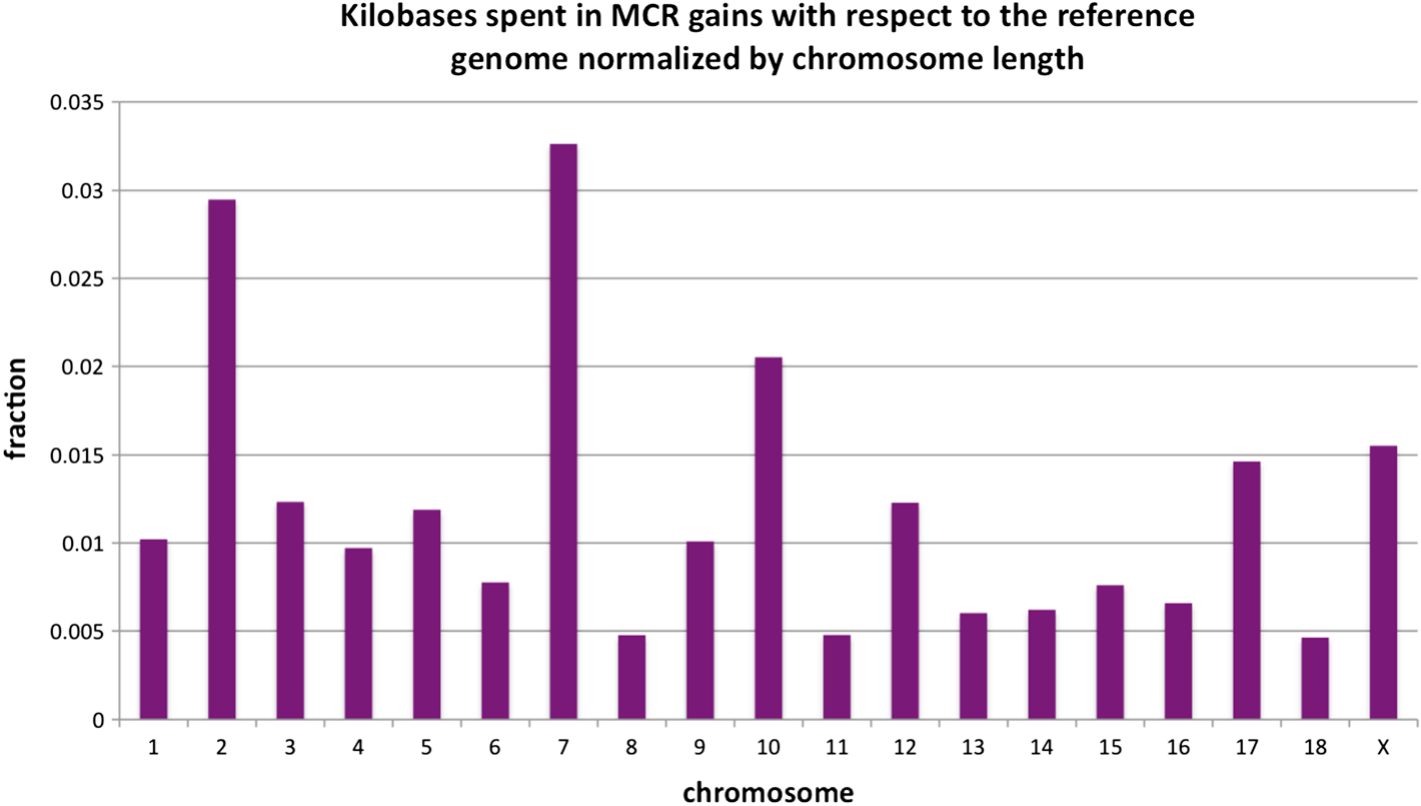
Total length of multicopy regions (MCR) per chromosome, divided by chromosome length, for each chromosome.

A total of 696 annotated genes fully fell inside MCRs and are therefore more likely to be functional than partially duplicated genes. Our study allowed us to discover novel paralogs of annotated genes, originally absent in the Duroc reference assembly. These genes primarily belonged to well-known multi-genic superfamilies. The most over-represented gene family by far was that of the olfactory receptors, comprising a total of 476 genes. The chromosomes containing the largest number of olfactory genes were SSC2 and SSC7 (Figure 6). These results agree with data from the international consortium, who found that the pig is one of the species with the largest repertoire of olfactory receptors, likely a result of the importance of scent in this foraging species [3]. Similarly, large gene families involved in defense and immune response were over-represented within MCRs; we found 8 new paralogs of annotated interferons (*IFN-α8, IFN-α10, IFNα-11, IFNα14, IFNδ2, IFNδ6, IFNω2* and *IFNω4*), 2 interleukines (*IL1-β, IL1B*) and five *SLA* genes (*SLA-3, SLA-9, SLA-10, SLA-P1, SLA-DRB1*). Several tumor necrosis factor receptors (*TNFR*) and T-cell receptors (*TR*) were found as well. Other genes within MCRs were involved in lipid (*ACOT4, GPAT2*) or carbohydrate metabolism (5 new paralogs of the *UGT2B* family and 8 salivary and pancreatic amylases), detoxification (*CYP2C33* and *CYP4A21*), pheromone binding (*PHEROA* and *PHEROC*), perception of taste (VN1R2) and fertilization (*SPM1*). Two genes from the serpin-like clade (Serpina 3–1 and Serpina 3–2), retinol dehydrogenase (*RDH16*), the myostatin gene (*MSTN*) and the lactase gene (*LCT*) also seem to be present in multiple copies in the pig genome. Several small RNAs were also detected: two rRNAs (5S ribosomal RNA and 5.8S ribosomal RNA), one snoRNA (*SCARNA6*), one snRNA (*U1*) and two miRNAs. A complete list of genes entirely inside MCRs is shown in Additional file 3. A gene ontology (GO) enrichment analysis of biological processes (see Materials and methods) found an over-representation of sensory perception of smell (adjusted P value = 2.06×10^-117^), response to virus (adjusted P value = 2.99×10^-06^) and xenobiotic metabolism process (adjusted P value = 1.55×10^-02^).

### Outlier regions and potential selection targets

A matter of intense research is the study of patterns of nucleotide variability in domestic species. Outliers in these patterns with respect to the standard neutral model can be due to selection and then reveal genes of socio – economic interest, as well as helping to understand the effects of domestication and of artificial selection in the genome [3]. A serious challenge is that selection does not result in a single obvious signal (e.g., a selective sweep) but rather in a diversity of manifestations that depend on intensity and age of selective process as well as on the demographic history of the population [15,17]. Here, we employed a number of tests that pinpointed a series of genome regions, potentially enriched in non-neutral genes. We also took advantage, where possible, of the simultaneous availability of pool and individual data. Despite the fact the Guadyerbas strain only represents one of the Iberian varieties [9], it is conjectured that the strongest selective sweeps will be shared across all Iberian strains.

First, we examined extreme windows in terms of low and high variability for the Guadyerbas and the pooled data (see Materials and methods). A total of 132 genes were annotated within the lowest variability windows (Additional file 3). A window in SSC1 was specifically enriched in interferon genes (*IFNE, IFN-α10, IFNω1, IFNω3* and *IFNω4*), which are involved in response to virus (adjusted enrichment GO P=1.3×10^-04^). Note that *IFN-α10* and *IFNω4* are within MCRs, suggesting that those genes have un-annotated paralogs and putatively under positive selection. Genes within the lowest variability windows in NPAR included genes from the Ras oncogene family (*RAB33A, RAB39B, RAB39B* and *RAP2C*), the *SOX3* gene, involved in sex determination, face development and pituitary gland development, the serotonin receptor *HTR2C*, involved in anxiety, reproductive and feeding behavior, the *MECP2*, with a role in behavioral fear response, as well as genes involved in lipid metabolism (i.e., *ACSL4, ALG13, ABCD1, PLP1*), in hair follicle development (*NSDHL*) and other genes related to immune response (*IL13Ra1, IL1RAPL2*). A complete list of these genes is in Additional file 3.

The majority (~80%) of the high variability windows contained MCRs identified in the individual sow as described above. To ensure that the high variability found is not influenced by MCR, we removed the SNPs inside MCRs. The result was that those windows still conserved high variability levels, in agreement with results in Table 4. The majority of genes in those windows were olfactory receptors, hundreds in total, present in gene clusters distributed among almost all chromosomes. In addition, other gene families were represented, e.g., *ATP*-binding cassette family, zing finger genes, T-cell receptors (*TR*) and *SLA* genes (mainly located in chromosome 7), transmembrane proteins (*TMEM* family), several small nucleolar RNAs, solute carrier family genes, protocadherin family genes involved in homophilic cell adhesion and cytochrome family p450 genes (*CYP*); see Additional file 3 for a complete gene list. Note that *IL1B* and other gene families are present in MCRs and also in high variability regions.

Next, we computed Tajima’s *D* and Fay-Wu’s *H* statistics, modified to account for the idiosyncrasy of pool data (Materials and methods). In principle, Tajima’s *D* and Fay-Wu’s *H* negative values can be produced by positive selection, although Tajima’s *D* is particularly sensitive also to demographic effects and prone to false positives. The correlation between both statistics was moderately positive r = 0.28 (Additional file 4). There are also an apparent number of windows with negative Tajima’s *D* and zero or even positive Fay-Wu’s *H*. Although the interpretation of this is not clear, it might be caused by recurrent hitch hiking events [18].

We selected the 1% most extreme windows with combined negative Tajima’s *D*, Fay-Wu’s *H* and low variability θ*w* (see Materials and Methods and Additional file 3 for full results). No over-representation of GO were detected after correcting by multiple testing. Interesting candidate genes inside those windows are involved in axonogenesis and synapsis (*FOXP1, LRRK2, EHMT2, RAB11A, TEKT5, IGF1R, UNC13C, CNTN1, COL9A2, AXIN2, CADPS2, HTR6, KCND1, NOVA1, PTEN*), circadian rhythm (*HEBP1, ALB*), epithelial cell differentiation, keratinization and hair follicle formation (*FOXP1, IGF1R, HNF1B, PTEN, AXIN2, KRT81, KRT83, KRT84, KRT85, KTR86, PRKD1, AC0210066.1*), blood vessel morphogenesis (*PPAP2B, PRKD1*), lipid metabolism and fat cell differentiation (*PPAP2B, VEPH1, RASA4B, ATP10B, NEU1, PTEN, SMPD4, ALB*), exploratory behavior (*LRRK2*), locomotory behavior (*APBA2*), grooming and feeding behavior (*NMUR2*), response to starvation (*GAS6, ALB*), spermatogenesis, ovulation and sex determination (*EHMT2, AFP, IGF1R*), visual/odor perception (*OR5P2, LRRK2, VSX1, GRK1*), immune response and inflammatory response (*CIITA, PRKD1, FOXP1, IGF1R, PTX3*). Importantly, the *LRRK2* gene is a positive regulator of the dopamine receptor signalling pathway. The complete gene list is in Additional file 3.

Finally, we performed a genomewide Hudson-Kreitman-Aguadé (HKA) test in the pool data. The NPAR was analyzed separately from autosomes and PAR. After correcting for multiple testing, only 25 windows (0.23%) with an excess of differentiation were significant (Benjamini-Hochberg [19] False Discovery Rate, FDR < 0.05). Genes within these windows were involved in feeding behavior (*NPW*), social behavior (*HTT, DVL1*), locomotory behavior (*HTT, SLCGA3*), pigmentation (*MC1R*), hair follicle morphogenesis (*PDGFA*), sensory perception of taste (*TAS1R3, GNG13*), perception of sound (*AXIN1*), circadian rhythm (*PRKAA2, ADCY1*), tumor necrosis factors (*TNFSF12A, TNFRSF18, TNFRSF4*), male gonad development and spermatogenesis (*GFER, BOK*), fat cell differentiation (*SDF4*), lipid metabolism (*DECR2*) and several genes in lipid transport, e.g., *ABCA3*, was also reported by the International Pig Genome Sequencing Consortium [3] being under selection. Interestingly, the neuropeptide *AXIN1* has been found differentially expressed in brains of two extreme groups of junglefow in terms of fearfulness [20]. The complete gene list is in Additional file 3.

Only 39 windows (0.36%) with an excess of polymorphism vs. differentiation were significant (HKA test False Discovery Rate < 0.05). Several genes inside those windows belonged to gene superfamilies (*ABC, OR, TRIM, Zinc fingers*). Interesting genes to mention are involved in immune response, e.g., complement system genes (*C8A, C8B*) and swine major histocompatibility complex (SLA-DQA1, SLA-DQB*G01, SLA-DRA1, SLA-DRB, SLA-DRB1), feeding behavior and synapsis (HCRTR2), visual/sound perception and pigment granule transport (*MYO7A*), lipid metabolism (*PPAP2A, PRKAA2*), viral infectious cycle (*RPS21*) or defense response (*SPACA3*) (full gene list in Additional file 3). Within the NPAR region of the X, only one window was significant. This window contains the *SHROOM2*, a gene involved in brain, eye and ear morphogenesis and pigment accumulation among others (Additional file 3).

## Discussion

This study presents a novel combined analysis of pool and individual sequencing. Although pools biases the SNP discovery process towards common variants and have lower power than individual sequencing [21], our simulation indicates that we should expect to detect almost half (47%) of all SNPs. Given that there are ∑ _*i* = 1,17_ 1/*i* = 3.4 times more SNPs in 18 chromosomes than in a single individual, the pool process uncovers about 60% more SNPs than individual sequencing – for any given region sequenced in common and assuming an average depth of 14× for the pool and 7x in the individual. Note that, nevertheless, the allele frequency spectrum is different. In pools, the SNP discovery is biased against low MAF SNPs, whereas the probability of a SNP being discovered in the individual is independent of its frequency in the population, assuming a neutral site frequency spectrum. The reason for this is that, although the probability of being heterozygous f(1-f) is maximum at frequency f=0.5 (high MAF), low MAF SNPs are much more common than high MAF SNPs and both effects cancel each other.

Genomewide variability in the Guadyerbas sow was much lower than that in the Iberian pool; 50% and 70% lower for autosomes and NPAR, respectively (Table 3). Estimates are corrected for the pooling process so the large disparity is not due to SNP calling in pools vs. individuals but, rather, to the high inbreeding of the Guadyerbas strain. Because the pedigree of the Guadyerbas is known since the foundation of the herd in 1944 [9], the inbreeding coefficient F for the specific sow sequenced was estimated from pedigree as F_A_ = 0.39 and F_X_ = 0.46 for autosomes and NPAR, respectively. This results in estimates corrected by inbreeding π_A*_ = 6.55×10^-4^ / (1–0.39) = 1.07×10^-3^ and π_X*_ = 1.79×10-4 / (1–0.49) = 3.51×10^-4^. These values are close to those obtained from the pool in autosomes but, intriguingly, for NPAR are still 40% lower in the Guadyerbas (Table 3). Therefore, inbreeding explains the loss in variability in the whole Iberian pig breed for autosomes but not in NPAR.

Remarkably, autosomal nucleotide diversity in the Iberian pool (0.0013, Table 3) are comparable to those reported in the two European wild boars sequenced by the International Pig Genome Sequencing Consortium: Heterozygosity He = 0.0012 and 0.0010 [3]. In contrast, heterozygosity in international domestic breeds is higher (He = 0.0016 or larger) than in European wild boar or Iberian pig, likely because of introgression with Asian pigs [22]. The fact that Iberian pigs and European wild boar diversities are comparable agrees with previous evidence showing that Iberian pigs have not been intercrossed with Asian germplasm [23]. It also stresses the relevance of Iberian pig as a model of native Mediterranean domestic pig that should help to disentangle the effects of Asian introgression and domestication on response to selection by modern breeding.

Our data further confirm the much lower observed than expected variability in SSCX (3/4 that of autosomes) as was previously reported in the partial resequencing of the same Guadyerbas sow [10]. Here, because we were able to distinguish between PAR and NPAR regions, the X/A ratio is even lower than reported before: 0.27 in Guadyerbas and 0.44 in the pool. In contrast, diversity in the PAR was comparable, or even higher, than in autosomes. Although demographic effects can reduce X/A variability, the effect observed here is quite unusual, and seems to be a pervasive property of several porcine populations [12]. Selection can be argued as an alternative explanation. Genes within the lowest variability NPAR windows included *ACSL4*, which is a candidate loci inside a QTL that affects fatty acid composition in the Iberian pig [24,25], *HTR2C*, which has been identified as a genetic loci potentially contributing to maternal infanticide in pigs [26], *SOX3*, which plays an important role in testis development and possibly sperm maturation [27], *MECP2* regulates fear-dependent learning and memory [28], a distinctive biological feature between wild animals and its domesticated descendants, *NSDHL* is involved in cholesterol biosynthesis but also in hair follicle formation, characteristic that has also evolved during domestication process, wild pigs are furrier than domestic pigs. It should be noted that the black varieties of Iberian breed, that include the Guadyerbas, are hairless and the red varieties present sparse hair.

The discovery of thousands of new MCRs (>4 kb) with respect to the reference genome potentially indicate many copy number variants between the Iberian pig and the Duroc reference assembly, although part of those could be due to a mis-assembled or incomplete reference genome in duplicated regions. In agreement with our results, Paudel et al., (op. cit) also report many new paralogs of existing genes in a diverse panel of pig breeds with respect to the reference Duroc assembly. The majority of them overlap with our results, except for *GPAP2*, *PHEROA*, *PHEROC* and *SPM1*, which might be Iberian specific MCR or only found here due to limited sampling in Paudel et al., (op. cit.). The fact that some MCRs have high values of nucleotide diversity might be caused by an artifact of the mapping (the Iberian pig presents more copies than the reference and therefore ambiguous reads map to the same locus, causing false positive SNPs). Nevertheless, the fact that variability in regions outside the MCR with respect to the assembly but within windows containing MCRs is higher than average genomewide (Table 4) might be an indirect consequence of increased recombination, which causes MCRs as well as increased variability. All in all, the precise interaction between recombination rate, variability and multicopy regions is currently conjecture.

To unravel putative genes under selection in the Iberian pig lineage we applied different selection tests operating at different time scales, primarily we focused on regions of very low variability, a combined, Tajima's *D*, Fay-Wu’s *H* and θ test and the HKA test. Some of the candidate genes found with extreme negative values of *D-H-θ* and low θ or excess of differentiation in the HKA test presented ontologies which have been previously reported to be under positive selection. Among those, genes related with keratinization and epidermis formation (*D-H-θ* test) have been reported to be under adaptive pressures in human and primates, they act as a physical barrier defense vis a vis the external environment [29-31]. Several studies in mammals and Drosophila have reported immunity related genes (evidence from θ*, D-H-θ* and HKA tests) as being under strong positive selection against rapidly evolving pathogens [32-37]. We also identified several genes involved in feeding behavior, fear response and social behaviour (*D-H-θ* and HKA tests). Behavior has been reported as one of the biological functions subject to selection during the process of pig domestication [12,38,39] and feeding behavior and response to starvation are, logically, most relevant traits in domestication and in breeding. Pigmentation (*MCR1* gene, HKA test), has been reported to be under positive selection in pigs due to human interest to cherry-pick different coat colors that would otherwise be quickly eliminated in the wild [40]. Spermatogenesis genes (*D-H-θ* and HKA tests) have been reported to be rapidly evolving genes in Drosophila and in many other organisms [41]. Finally, lipid metabolism genes (*D-H-θ* and HKA tests) might also have changed, specifically in the Iberian breed, conferring its distinctive lipid composition and deposition in the meat.

Finally, an excess of polymorphism in the HKA test or extreme high values of θ are indicative of balancing selection but could also be the result of artifacts due to assembly errors in duplicated regions. Given the widespread occurrence of MCRs reported here, further investigations in this direction are needed. In particular, improving the reference pig assembly, will help to disentangle both effects on polymorphism data. As reported in previous studies [42-44], genes involved in the perception of smell (olfactory receptor family*)* and genes involved in antigen presentation and defense response (e.g., *SLA)* are inside these regions.

## Conclusion

The recent completion of the porcine sequencing project has allowed digging deeper into the complexities of the Iberian pig genomes than was possible until now. This breed is important because it represents a primigenious European breed that, while being domestic, has not been introgressed with Asian germplasm. Our data confirm the importance of structural variation in the porcine species, as also observed in other species. The tests applied suggest that many and diverse selective processes have occurred in the Iberian pig lineage, among them changes in feeding behavior. New bioinformatics tools, e.g. that deal with structural variants, as well as projects aimed at complete annotation of the pig genome (ENCODE) are needed to improve interpretation of the results.

## Material and methods

### Samples and sequencing

The genome of a highly inbred Iberian pig, pertaining to the Guadyerbas strain, which has been partially sequenced (1% of the genome) in a previous study [10], was shotgun sequenced using Illumina Hiseq2000. We run one 100 bp PE lane and one 100 bp SE lane. In addition, we also sequenced a reduced representation library (RRL) of a pool comprising nine sows (equal concentrations of each) from the most representative Iberian varieties in Spain: *Retinto, Mamellado, Torbiscal, Guadyerbas, Entrepelado* and *Lampiño*. All sequenced sows are registered in the Iberian Herd Book and were sampled from well accredited farms that have kept purebred Iberian pigs without intercrossing with ‘foreign’ breeds. The method to construct the reduced representation library is described elsewhere [10]. For the pool, Illumina GAIIx technology of 50 bp was employed, and 2 PE lanes were available. As outgroup, we shotgun sequenced a *Potamocherus porcus* male using Hiseq2000 (three PE lanes, 100 bp long) in order to measure divergence and asses ancestral alleles so that we can apply more powerful tests to detect selection (HKA, Fay-Wu’s *H*).

We were able to delineate the boundaries between PAR and NPAR because of read depth differences in males along SSCX (unpublished data). The SSCX PAR occupies the first 6.7 Mb and the last 400 kb of SSCX, approximately. Although assembly 10.2 separates two telomeric PARs, linkage analyses using genotyping data from the 60k SNP chip in an Iberian x Landrace cross and results from Burgos-Paz [45] suggest that a single PAR exists – as in most mammals. We therefore pooled the results from the two annotated PARs in the analyses reported here.

### Alignment and SNP calling

Reads were mapped against the latest reference genome (assembly 10.2) using BWA [46], allowing 7 mismatches and filtering by mapping quality of 20. *P. porcus* reads were aligned disregarding the paired end structure, i.e., they were aligned as SE. This was done to minimize the possibility that structural changes between the two species prevent alignment. A total of 345M reads were aligned, resulting in an average depth of 20× (3-50×) and 1.6 GB of the *S. scrofa* genome assembled.

SNP calling for the Guadyerbas individual was performed using samtools mpileup option [47] filtering by minimum depth of 3×, maximum depth of 20× and SNP quality of 20. SNP calling in the Iberian pool was done using SNAPE (http://code.google.com/p/snape-pooled/) [48], setting divergence to 0.01, prior nucleotide diversity 0.001, folded spectrum and filtering by a posterior probability of segregation > 0.90. The SNAPE approach consists in computing the posterior probability of SNP frequency being distinct from 0 or 1, given that we observed *n*_*A*_ alternative alleles and *C-n*_*A*_ reference alleles, and given prior frequency in the population being P(*f*):

$$P\;\left(f/n_{A}\right)\propto P\left(n_{A}/f\right)\;P\left(f\right)$$

Where

$$P\left(n_{A}/f\right)=\sum_{k=0}^{n}{(}_{n_{A}}^{C})p^{n_{A}}{\left(1-p\right)}^{C-n_{A}}{(}_{k}^{n})f^{k}{\left(1-f\right)}^{n-k},$$

with *p* being the probability that an allele A is read and *n,* the number of chromosomes in the pool. This probability in turn depends on *n*, *k* and on whether there is a true A in the genome or whether it is the result of a sequencing error. The algorithm considers the geometric mean of sequence qualities for every allele read to compute this probability [48]. In the equation above, we take into account the probability that *k* counts of the allele are present in the pool, given that its true frequency is *f* and that, given *k*, how many reads A out of *n* are expected. Because some quantities, notably *k*, is unknown, this is integrated out. For prior p(*f*), we considered the standard neutral model expected frequency, i.e., *f* α 1/*f*.

### Simulation of pooling process

Although pools are a highly cost-effective strategy, the variability uncovered is only a fraction of the true variability in the population. We sought to evaluate the power and false discovery rate of our experimental design by simulation. We simulated 18 chromosomes of 1 Mb using coalescence with ms program [49] under a standard neutral model with nucleotide diversity π and scaled recombination rate ρ per site = 0.001. For each resulting chromosome, the program ART [50] was used to generate reads with the built-in profile for Illumina paired-end technology of 75 bp-long reads. To simulate the pooling process, reads were randomly selected from each sequence using an equal proportion from each individual. An average depth of 14× was simulated for the whole pool in all and reads were aligned with BWA [46]. Next, SNPs were called with SNAPE, restricting minimum and maximum depths to do the calling between 3× and 30× as in our real data analyses. Power was computed as the proportion of true SNPs in the population (i.e., before pooling) located within regions of appropriate depth that were correctly recovered. False Discovery Rate (FDR) was the proportion of SNP calls that were incorrect. A total of 100 replicates were simulated.

### Multicopy region detection

Read depth method [51,52] was applied to identify copy number of a region. Basically, we employed the same pipeline as in Paudel et al (op. cit., submitted). First, we employed mrsFAST [53], an exhaustive mapping tool that allows paralog detection, to align reads (allowing 6 mismatches) against the repeat masked reference genome; repeat mask information was obtained from NCBI. Average read depth for each non-overlapping 1kb bin was calculated across the genome and copy number (CN) of each unit was predicted based on the average read depth across the diploid region. 1:1 orthologous genes between human, cow and pig was used to obtain read depth across diploid region. Since these regions have the same number of copies in 3 relatively distant species, we assumed these were conserved in a copy number neutral stage. Finally, chained regions in the genomes which are ≥ 4kb in length having copy number ≥3 (each bin should have CN ≥ 3 and 1 kb gap was allowed), were extracted and declared MCRs. Next generation sequencing methods introduce bias in the read depth, which is caused by the dissimilar GC content of different segments of DNA. To correct this bias, we used GC intervals and the average read depth across the diploid region to find out the correction factor and used that factor to correct depth of each 1 kb bins [52].

### Nucleotide variability estimation and selection tests

Note that, with next generation sequencing data at low depth, nucleotide diversity cannot be simply computed dividing the number of SNPs called by the length of sequence assembled. This is because, with shallow depth, the two alleles of the same SNP may not be read and because of errors in calling SNPs. For the individual, we corrected for low coverage as detailed in [10]:

$${\hat{\theta }}_{w}=\frac{S}{\sum_{i}L\left(i\right)P\left(j\backslash i\right)}$$

where *S* is the raw number of SNPs, *L(i)* is the length in bp of depth *i* for that window, and *P(S|i)* is the probability of reading both alleles for depth *i*[10]. In the case of pools, Watterson’s theta was computed as in [12]. Briefly, we correct by the expected number of chromosomes sampled for each read depth along the window:

(1) $${\hat{\theta }}_{w}=\frac{s}{\sum_{i}L\left(i\right)\sum_{j=2}^{\min\left(\mathrm{nr}\left(i\right),\mathrm{nc}\right)}P_{c}\left(j\backslash \mathrm{nr}\left(i\right),\mathrm{nc}\right)\;a_{j}}$$

where *L(i)* is the length in bp of depth *i* for that window, and Pc( *j* | *nr(i), nc*) is the probability that a set of *nr* sequences randomly extracted from *nc* possible chromosomes contains sequences coming from precisely *j* different chromosomes. Finally, *a*_*j*_ is Ewens constant ∑ _*i* = 1,*n* − 1_1/*i*.

### Definition of low and high variability windows

Given that over 10% of Guadyerbas windows had no SNP, we defined extreme low variability regions for the Guadyerbas as those windows devoid of variability and with > 10kb assembled. Among these windows, we selected those of 5% lowest variability in the pool as well, with a minimum of 3 kb aligned. In that way, we avoid choosing fixed regions in the Guadyerbas strain due to drift. We defined extreme high variability regions as the 5% most variable windows in Guadyerbas and in the pool where at least 10 kb (Guadyerbas) and 3 kb (pool) were aligned.

### Tajima’s D and Fay-Wu’s H tests

Tajima’s D test [54] were computed as the normalized difference between the average pairwise nucleotide difference θπ and the Watterson estimator, divided by the theoretical variance of the same difference in the standard neutral model without recombination in pools (Ferretti L, Ramos-Onsins SE, Pérez-Enciso M: Population genomics from next generation sequencing of pooled lineages, submitted). The estimator of θ based on π was computed as the average pairwise nucleotide diversity across all reads for a given position, averaged over all positions and corrected by a multiplicative factor 2n/(2n-1) [55]. This estimator is unbiased under the neutral model. The normalized Fay and Wu’s H test [56] was computed similarly from the standardized difference between θπ and the estimator θ_H_ based on high frequency derived alleles. For the estimator θ_H_, only sites with known outgroup bases were used, and the estimator was obtained by summing all segregating sites with *k* derived alleles in *r* reads weighted by the factor k^2^/r(r-1) and divided by a factor correcting for the bias (Ferretti et al., op cit.). The variances in the denominators are evaluated exactly in the limit of short read for the standard neutral model without recombination following the results of [57] and accounting for the random extraction of reads from individuals. Code is available from L. Ferretti (luca.ferretti@gmail.com).

In order to minimize confounding demographic effects with selection fingerprints, we calculated the empirical joint distribution combining Tajima's *D*, Fay and Wu's *H* and Watterson’s θ as in [58]. To do so, we sorted the normalized statistics D, H and θ, the empirical test was obtained simply by multiplying the inverse of the ranks 1/*M*, 2/*M*, … 1 of each statistic for each window 1 … *M*, and normalizing. A GO enrichment analysis was performed with genes within the 1% most extreme windows.

### Hudson-Kreitman-Aguadé test

Multilocus Hudson-Kreitman-Aguadé (HKA) tests were calculated in the pool using the *P. porcus* alignment as outgroup and following the original algorithm [59]. We applied the test dividing the genome in 200 kb windows. Then, M+1 equations were solved using a bisection algorithm to calculate the estimates of the M+1 parameters (M theta values, one per window, plus the time of split between species measured in 2Ne generations). Thus, a partial HKA test per window was obtained plus the total sum of values, where the null hypothesis (stationary neutral model) is contrasted using M-1 d.f. The approach assumes unlinked windows and it is, therefore, conservative because nearby windows are linked. The original HKA formulae require a_n_ = ∑ _*i* = 1,*n* − 1_1/*i* and b_n_ = ∑ _*i* = 1,*n* − 1_1/*i*^2^ constants, which in the case of pooling are unknown. Instead we used the equivalent correction to infer Watterson’s theta from pools (denominator in eq. 1), whereas bn was obtained by interpolation from a_n_. The HKA function can be downloaded from http://bioinformatics.cragenomica.es/numgenomics/people/sebas. In order to identify outlier windows we performed a Benjamini-Hochberg [19] multiple test correction over the value of the partial Chi-square per window using a 5% false discovery rate.

### Annotation and Gene Ontology enrichment analysis

SNP annotation was performed using the Variant Effect Predictor perl script from Ensembl [11] and the *Sus scrofa* gtf annotation file was from Ensembl release 67, the latest version and that used in the pig genome publication. Gene ontology enrichment analysis was performed using FatiGO, a module of Babelomics using the human genome as background and converting Ensembl pig IDs to Ensembl human IDs.

### Data accessibility

Aligned reads in bam format are accessible at sequence read archive (SRA), http://www.ncbi.nlm.nih.gov/sra (experiment ID: SRX245748).

### Ethics statement

Animal manipulations were performed according to the Spanish Policy for Animal Protection RD1201/05, which meets the European Union Directive 86/609 about the protection of animals used in experimentation.

## Abbreviations

CYP: Cytochrome P450 family; GO: Gene ontology; HKA: Hudson-Kreitman-Aguadé; IL: Interleukine; INF: Interferon family; MAF: Minor allele frequency; MCR: Multicopy region; NPAR: Non-pseudoautosomal region; OR: Olfactory receptors; PAR: Pseudoautosomal region; PE: Paired-end; RRL: Reduced representation library; SE: Single-end; SLA: Swine leukocyte antigens; TNF: Tumor necrosis factor; TR: T-cell receptors.

## Competing interests

The authors declare that they have no competing interests.

## Authors' contributions

AEC and MPE analyzed data. ER, LF, SERO, YP, HJM, and MAMG provided analytical tools and helped in the analyses. LS and MCR provided material. AEC and MPE wrote the manuscript with help from the rest of authors. MPE conceived and coordinated research. All authors read and approved the final manuscript.

## Supplementary Material

Additional file 1 ** Simulated power against depth.** Power was computed as the number of SNP called by SNAPE software divided by the total number of real SNPs in the pool. Depth corresponds to the average depth in the pooled data. Bottom: Power against MAF (minor allele frequency in the pool). Click here for file

Additional file 2 Variability (Wattersons's estimate, per bp) inside multicopy regions vs. variability of windows containing multicopy regions but outside the multicopy region units? Click here for file

Additional file 3 **Genes within multicopy regions and extreme selection tests’ windows.** MCR genes: genes within multicopy regions; Lowest theta shared autosomes: genes within extreme low θ in autosomes and X pseudoautosomal region (PAR) common in the individual and the pool; Lowest theta shared non-pseudoautosomal region (NPAR): genes within extreme low θ in X NPAR region common in the individual and the pool; Largest theta pool: genes within extreme high θ regions in the pool; Largest theta individual: genes within extreme high θ regions in the individual; Lowest combined test: genes with lowest values of the combined Tajima’s *D*- Fay&Wu’s *H* and θ test; ΗΚΑ excess of differentiation autosomes+PAR: genes within HKA excess of differentiation in autosomes and X PAR region; ΗΚΑ vexcess of polymorphism autosomes+PAR: genes within HKA excess of polymorphism in autosomes and X PAR region; ΗΚΑ excess of polymorphism NPAR: genes within HKA excess of polymorphism in X NPAR region. Click here for file

Additional file 4 Correlation across 200 kb windows between Tajima’s D and Fay - Wu’s H statistics in pooled data. Regression line is shown in red. Click here for file
