# Supplementary figures and images for: Dissecting structural and nucleotide genome-wide variation in inbred Iberian pigs
Source: BMC Genomics. 2013 Mar 5;14:148. doi: 10.1186/1471-2164-14-148 (PMC3601988; doi:10.1186/1471-2164-14-148)

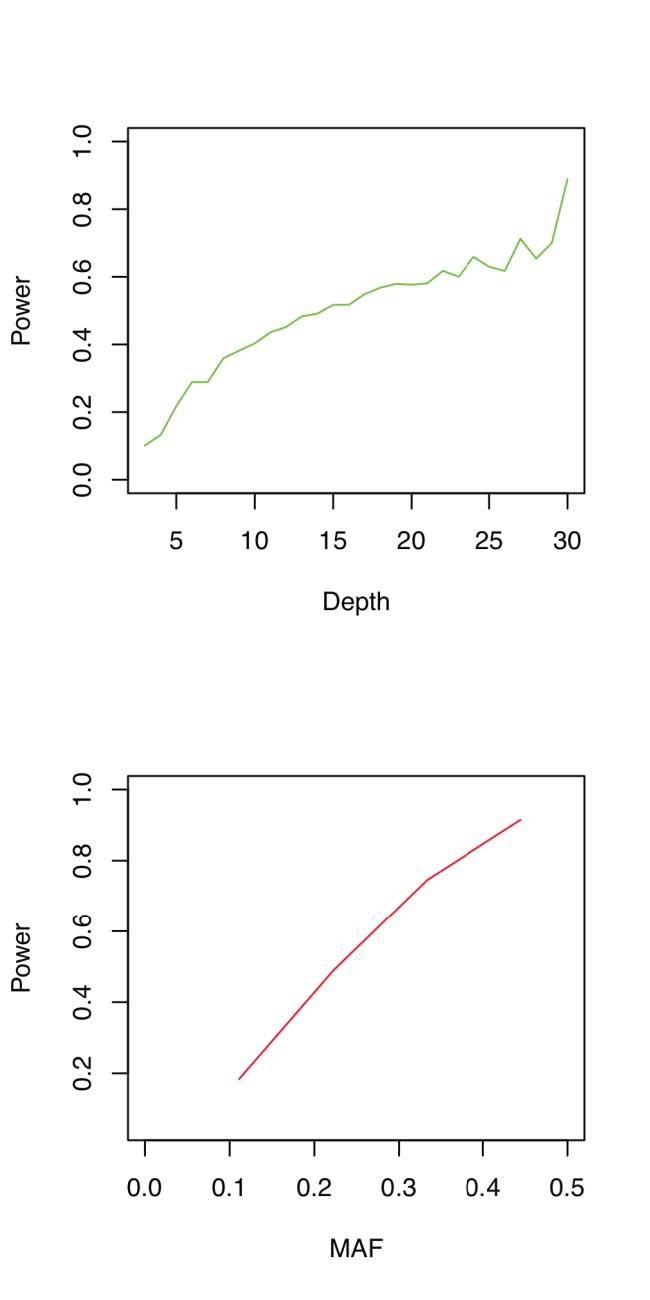

Supplement: Additional file 1 — Simulated power against depth. Power was computed as the number of SNP called by SNAPE software divided by the total number of real SNPs in the pool. Depth corresponds to the average depth in the pooled data. Bottom: Power against MAF (minor allele frequency in the pool). [file 1471-2164-14-148-S1.jpeg]

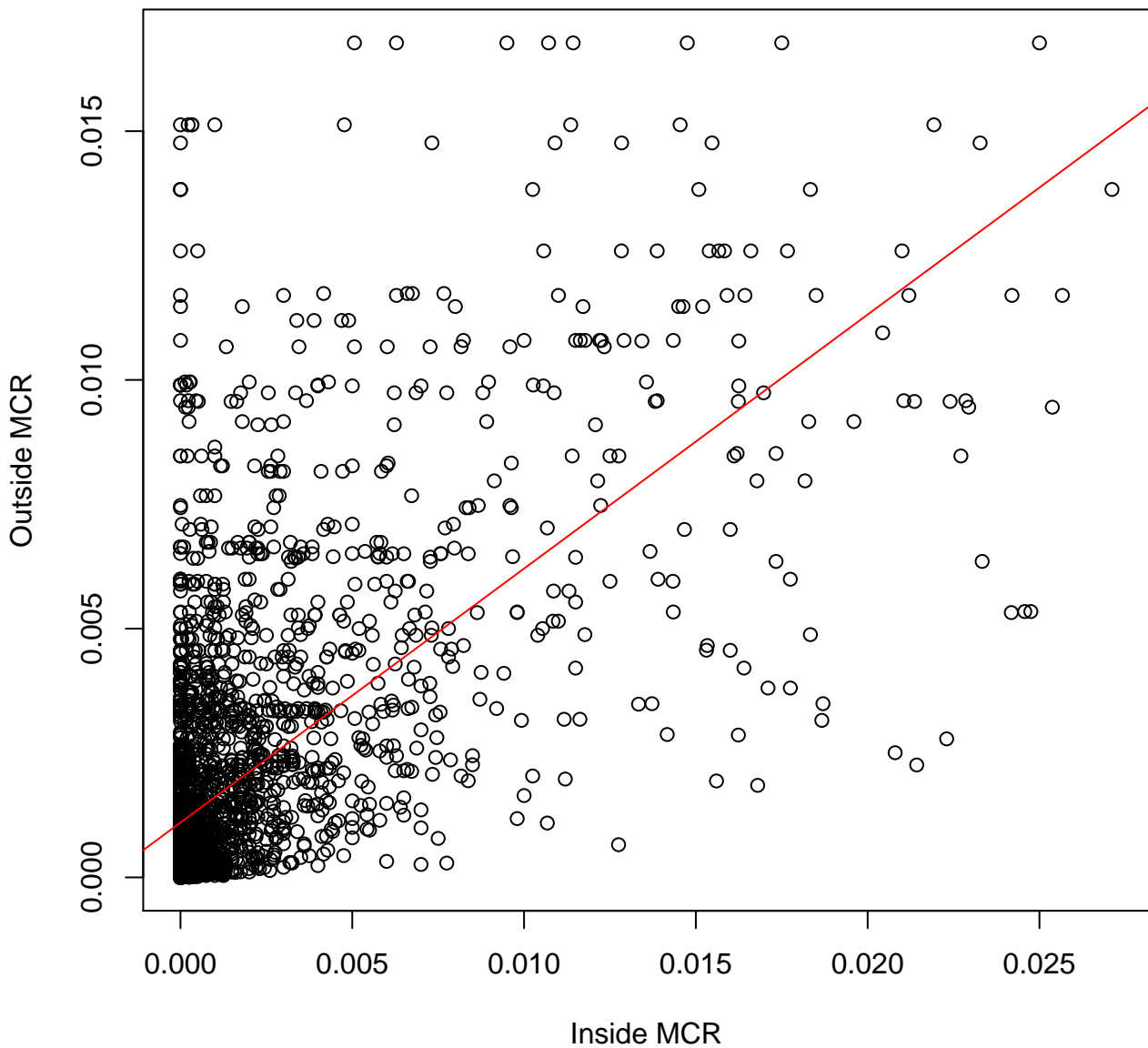

Supplement: Additional file 2 — Variability (Wattersons's estimate, per bp) inside multicopy regions vs. variability of windows containing multicopy regions but outside the multicopy region units? [file 1471-2164-14-148-S2.pdf]

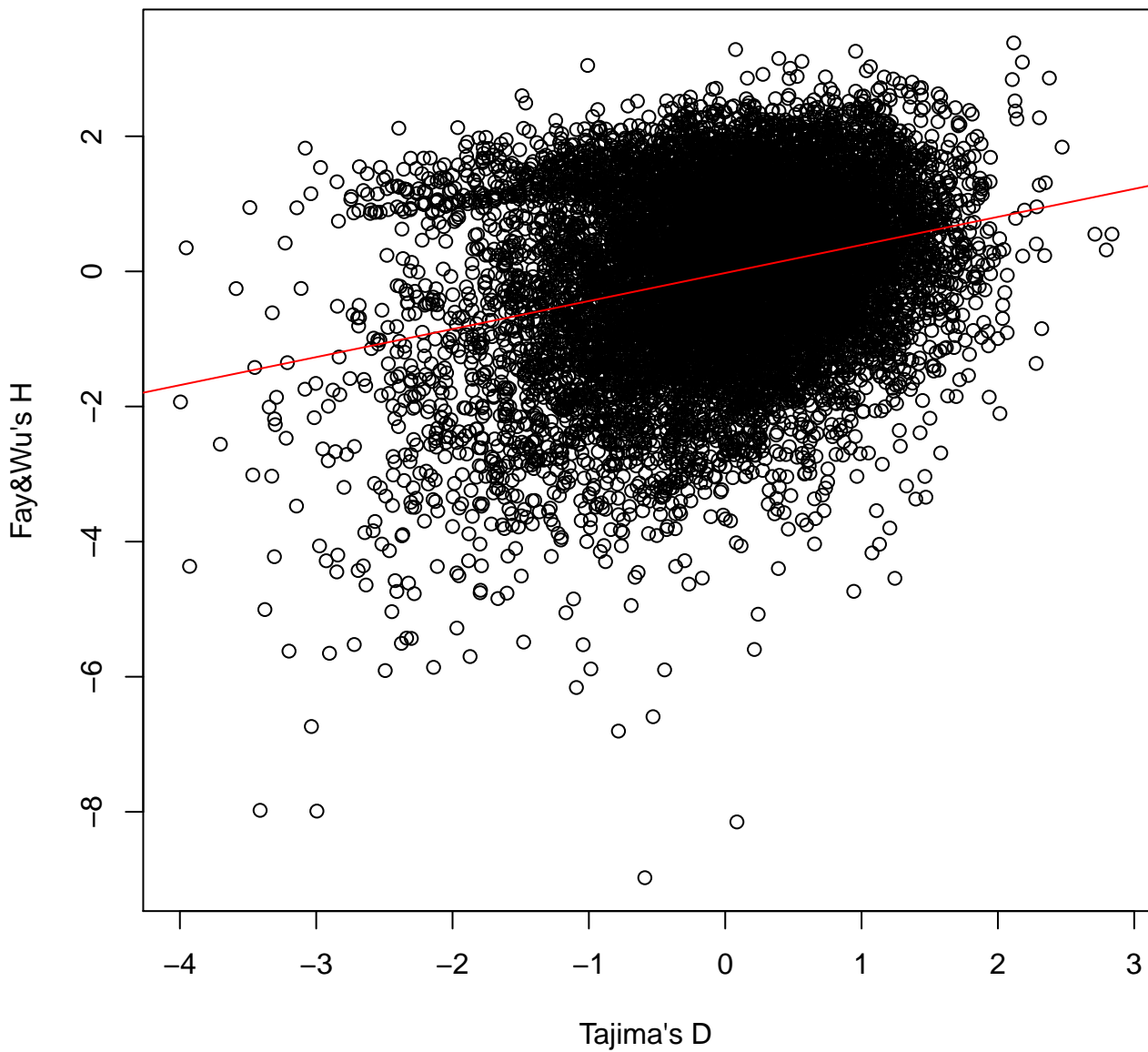

Supplement: Additional file 4 — Correlation across 200 kb windows between Tajima’s D and Fay - Wu’s H statistics in pooled data. Regression line is shown in red. [file 1471-2164-14-148-S4.pdf]
